# Fish Oil Containing Pro-Resolving Mediators Enhances the Antioxidant System and Ameliorates LPS-Induced Inflammation in Human Bronchial Epithelial Cells

**DOI:** 10.3390/ph17081066

**Published:** 2024-08-14

**Authors:** Alfio Distefano, Laura Orlando, Sebastiano Giallongo, Emanuela Tropea, Mariarita Spampinato, Annalisa Santisi, Lucia Longhitano, Giuseppe Parisi, Salvatore Leonardi, Arcangelo Russo, Massimo Caruso, Michelino Di Rosa, Daniele Tibullo, Maurizio Salamone, Giovanni Li Volti, Ignazio Alberto Barbagallo

**Affiliations:** 1 Department of Biomedical and Biotechnological Sciences, University of Catania, 95123 Catania, Italy; distalfio@gmail.com (A.D.); lauraorlando2810@gmail.com (L.O.); tropeaemanuela3@gmail.com (E.T.); mariaritaspampinato93@gmail.com (M.S.); annalisa_santisi@hotmail.it (A.S.); lucia.longhitano@unict.it (L.L.); mascaru@unict.it (M.C.); mdirosa@unict.it (M.D.R.); daniele.tibullo@unict.it (D.T.); msal37@gmail.com (M.S.); livolti@unict.it (G.L.V.); ibarbag@unict.it (I.A.B.); 2 Department of Medical, Surgical Sciences and Advanced Technologies “G.F. Ingrassia”, University of Catania, 95123 Catania, Italy; 3 Pediatric Respiratory Unit, AOUP “G. Rodolico-San Marco”, University of Catania, 95123 Catania, Italy; gf.parisi@policlinico.unict.it (G.P.); leonardi@unict.it (S.L.); 4 Department of Medicine, Kore University of Enna, 94100 Enna, Italy; arcangelo.russo@unikore.it

**Keywords:** lung disease, omega-3, PUFAs, resolvins, pro-resolving mediators

## Abstract

Fish oil, renowned for its high content of omega-3 fatty acids, particularly eicosapentaenoic acid (EPA) and docosahexaenoic acid (DHA), has gained considerable attention for its potential health benefits. EPA and DHA exhibit anti-inflammatory effects by promoting the production of specialized pro-resolving mediators (SPMs), such as resolvins and protectins. Fish oil has been studied for its potential to reduce bronchial inflammation, a key feature of respiratory conditions like asthma and COPD. This study investigates the cellular mechanisms of fish oil in an in vitro model of lung inflammation using lipopolysaccharide (LPS) on a healthy human bronchial epithelium cell line. LPS exposure for 24 h reduced cell viability, elevated reactive oxygen species (ROS), depleted glutathione (GSH), and induced mitochondrial depolarization, indicating oxidative stress and inflammation. Fish oil administration significantly mitigated ROS production, prevented GSH depletion, and reduced mitochondrial depolarization. This was associated with the upregulation of the endogenous antioxidant system, evidenced by restored GSH levels and the increased gene expression of glutathione peroxidase (GPX), catalase (CAT), superoxide dismutase 1 (SOD1), and superoxide dismutase 2 (SOD2). Fish oil also suppressed IL-6 and IL-1β expression and increased anti-inflammatory cytokine IL-10 expression. Furthermore, fish oil upregulated the expression of pro-resolving mediator receptors, suggesting a role in inflammation resolution. These findings highlight the potential of fish oil supplementation as a preventive measure against pulmonary diseases characterized by unresolved inflammation such as lung inflammation.

## 1. Introduction

Fish oil (FO) has been widely used for the treatment of different diseases given the evidence rising from clinical, experimental, and epidemiological observations. Its properties are mainly given by the omega-3 fatty acids and show therapeutic properties against those diseases characterized by an inflammatory status. Omega-3 fatty acids, particularly eicosapentaenoic acid (EPA) and docosahexaenoic acid (DHA), have been extensively studied for their ability to modulate inflammatory processes and oxidative stress. These fatty acids are known to reduce the production of pro-inflammatory cytokines and reactive oxygen species (ROS), thereby ameliorating inflammation and oxidative damage in various tissues, including the lungs [1]. The underlying mechanisms involve the activation of key signaling pathways such as the nuclear factor erythroid 2–related factor 2 (Nrf2) pathway, which plays a critical role in the cellular antioxidant response and is involved in the expression of pro-inflammatory genes, thereby reducing the production of inflammatory cytokines such as TNF-α, IL-1β, and IL-6 [2].

In the context of lung diseases, the beneficial effects of omega-3 fatty acids have been demonstrated in several studies. For instance, dietary fish oil has been shown to alleviate symptoms and improve lung function in models of chronic obstructive pulmonary disease (COPD), asthma, and acute respiratory distress syndrome (ARDS) [3,4,5]. These effects are attributed to the anti-inflammatory and antioxidant properties of EPA and DHA, which help to reduce oxidative stress and inflammation in the respiratory system.

The human body can synthesize EPA and DHA from alpha-linolenic acid through a series of elongation and desaturation steps. However, this biosynthesis pathway is relatively inefficient, necessitating direct intake from dietary sources or supplements to achieve optimal levels [6]. As a result, fish oil supplements have become a popular and efficient means of increasing omega-3 intake, leading to a surge in their use in recent years [7,8].

**Figure 1:**
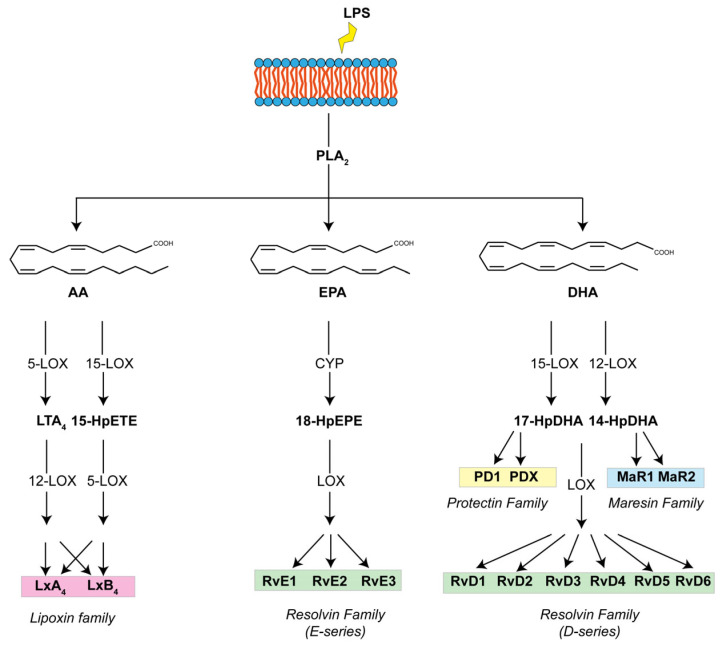
Biosynthetic pathways of specialized pro-resolving mediators (SPMs). Lipoxins (LxA4 and LxB4) are produced from AA, E-series of resolvins (RvE1, RvE2, and RvE3) are produced from EPA, and D-series of resolvins (RvD1, RvD2, RvD3, RvD4, RvD5, and RvD6), protectins (PD1 and PDX), and maresins (MaR1 and MaR2) are produced from DHA. HpEPE: hydroperoxyeicosapentaenoic acid; HpDHA: hydroperoxydocosahexaenoic acid.

Numerous studies have revealed pro-resolving mediators (SPMs) derived from omega-3 and 6-polyunsaturated fatty acids (PUFAs) that include arachidonic acid lipoxins, eicosapentaenoic acid (EPA)-derived (E-Series) and docosahexaenoic acid (DHA)-derived resolvins (D-Series), maresins, and protectins, which have various roles in promoting the resolution of inflammation and regulating the immune system [9,10,11,12] (Figure 1). 

SPMs are not present in the diet, are endogenously produced during inflammation, have potent anti-inflammatory actions, and are able to promote the resolution of inflammation [13].

SPMs act to reduce the neutrophil recruitment of pro-inflammatory cytokines. In humans, fish oil is an important source of EPA and DHA, and its supplementation has been found to increase circulating levels of many SPMs [14]. Many studies concerning the effects of EPA, DHA, and their SPM on in vivo and in vitro models of respiratory disorder demonstrated that EPA and DHA have a positive influence in mitigating the pro-inflammatory response in respiratory diseases and reduction symptoms, such as nasal congestion, difficulty in breathing, and fever [15,16].

These disorders are characterized by persistent inflammation and oxidative stress, which contribute to progressive lung tissue damage and impaired respiratory function [17,18]. Recent studies have highlighted the potential of dietary interventions, particularly those involving omega-3 polyunsaturated fatty acids (PUFAs), to mitigate these deleterious effects [19]. So, among omega-3 PUFAs, EPA and DHA, primarily sourced from fish oil, have garnered significant attention for their anti-inflammatory and antioxidant properties [20].

Therefore, our study aims to elucidate the cellular mechanisms by which fish oil exerts its protective effects in an in vitro model of lung inflammation. For this purpose, we used lipopolysaccharide (LPS)-induced inflammation in a human bronchial epithelial cell line to investigate the impact of fish oil on oxidative stress, mitochondrial function, and the expression of key antioxidant and inflammatory markers. This research seeks to provide a deeper understanding of how omega-3 fatty acids can be harnessed to develop novel therapeutic approaches for managing chronic inflammatory lung diseases.

## 2. Results

### 2.1. FO Exerts a Protective Role against LPS-Induced Cell Death

**Figure 2:**
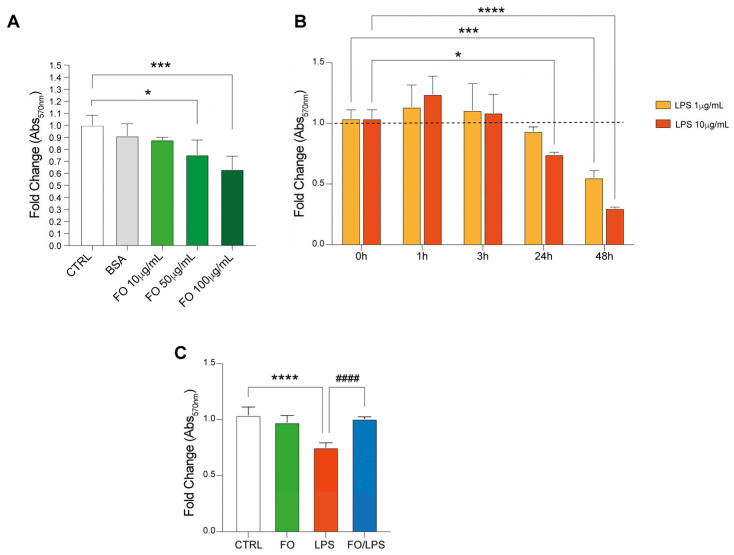
FO and LPS impact on cell viability. (**A**) FO supplementation at 100 mg/mL decreases BEAS viability. MTT assay quantification detecting cell viability following FO treatment at 10, 50, and 100 μg/mL. BSA condition was included given its role as FO vehicle. (**B**) LPS supplementation hampers BEAS viability following 24 h treatment. Histograms are representative of MTT assay performed on BEAS treated with 1 and 10 μg/mL LPS at different timepoints. (**C**) FO supplementation recovers LPS-induced cell death. Quantification of MTT test assessing cell viability following FO 10 μg/mL, LPS 10 μg/mL single treatments, and their combination. Histograms are representative of 4 biological replicates. (* *p* ≤ 0.05; *** *p* ≤ 0.001; **** *p* ≤ 0.0001 compared to CTRL. #### *p* ≤ 0.0001 compared to LPS).

To establish its best dosage in our in vitro model, we decided to perform an MTT assay supplementing FO for 24 h at concentrations of 10, 50, and 100 μg/mL. Of note, the impact of 1% BSA, used as a vehicle in our model, was also included in this test. Our results show no significative decrease in cell viability following either BSA or 10 μg/mL FO supplementation (Figure 2A). Interestingly, we detected a 60% and 70% cell viability following, respectively, 50 and 100 μg/mL dosages in our model (Figure 2A). 

In order to evaluate the effectiveness of FO treatment in attenuating inflammation, we also decided to supplement two different dosages of LPS at different timepoints in our in vitro model. It is well established indeed that, once triggered, inflammation might lead to cell death. In this context, necrosis is currently considered as the main form of cell death caused by inflammation [21]. Interestingly, our MTT assay showed a marked decrease in cell viability following 24 h LPS treatment at 10 μg/mL in comparison to T0 and to untreated counterparts (Figure 2B, dotted line). A significative hampering of cell viability at 1 μg/mL, on the other hand, was detected only after 48 h (Figure 2B).

Therefore, to evaluate if FO supplementation might attenuate LPS-mediated cell death, we decided to pretreat BEAS with a sublethal dosage of 10 μg/mL FO for 24 h and subsequently supplement LPS 10 μg/mL for the next 24 h (Figure 2C). Interestingly, our data show a significative decrease in cell death in the FO-pretreated condition in comparison to LPS-supplemented cells, eventually showing significative changes compared to the untreated counterpart (Figure 2C). 

Taken together, these data allowed us to detect an FO-mediated attenuation of LPS-induced cell death.

### 2.2. FO Supplementation Mitigate LPS-Induced Oxidative Stress

**Figure 3:**
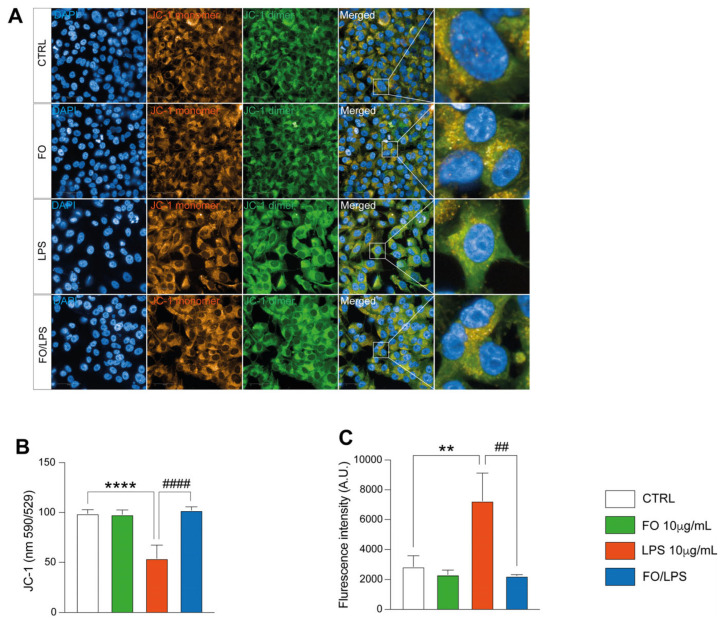
FO supplementation recovers cells’ LPS-induced mitochondrial potential loss and oxidative stress. (**A**) FO supplementation prevents LPS-induced JC-1 signal decrease. Representative images showing JC-1 assay in BEAS, supplemented with FO, LPS, and their combination. (**B**) FO recovers LPS-induced mitochondrial depolarization. Quantification of JC-1 signal as in (**A**). Histogram showing JC-1 quantification following BEAS treatment with FO, LPS, and their combination. (**C**) FO restores LPS-induced oxidative stress. Histogram showing ROS quantification by fluoresceine fluorescence measurement following BEAS treatment with FO, LPS, and their combination. Histograms are representative of 4 biological replicates. (** *p* ≤ 0.01; **** *p* ≤ 0.0001 compared to CTRL; ## *p* ≤ 0.01; #### *p* ≤ 0.0001 compared to LPS).

Inflammation, as one of the main outcomes, triggers the accumulation of reactive oxygen species (ROS), eventually leading to mitochondrial loss of function [22,23]. To assay if FO might recover this outcome, we sought to assess mitochondrial polarization by using a JC-1 probe (Figure 3A,B). Interestingly, immunofluorescence data proved that FO per se did not impair mitochondrial fitness, as the JC-1 signal does not show any significant change in comparison to the untreated counterpart (Figure 3A,B). LPS supplementation, on the other hand, marked a significative decrease in mitochondrial polarization, as depicted by the significative depression of the JC-1 signal in comparison to both untreated and FO counterparts (Figure 3A,B). Most importantly, FO pretreatment avoids LPS-mediated decreases in mitochondrial polarization, recovering the JC-1 signal to a level comparable to CTRL (Figure 2A,B), suggesting one of the mechanisms by which FO avoids cellular death.

To further support these data, we also assessed ROS accumulation in BEAS extracellular media by assaying fluoresceine fluorescence intensity. Interestingly, our data show that LPS supplementation, but not FO, dramatically increases the fluoresceine signal, overall indicating an enhancement of ROS levels (Figure 2C). Interestingly, once pretreated with FO, LPS could not exert its pro-inflammatory role, as shown by the significant decrease in the fluoresceine signal in comparison to the LPS experimental condition. 

Overall, these data unveil a crucial effect in avoiding LPS-mediated oxidative stress in FO.

### 2.3. FO Triggers Antioxidant Defenses

**Figure 4:**
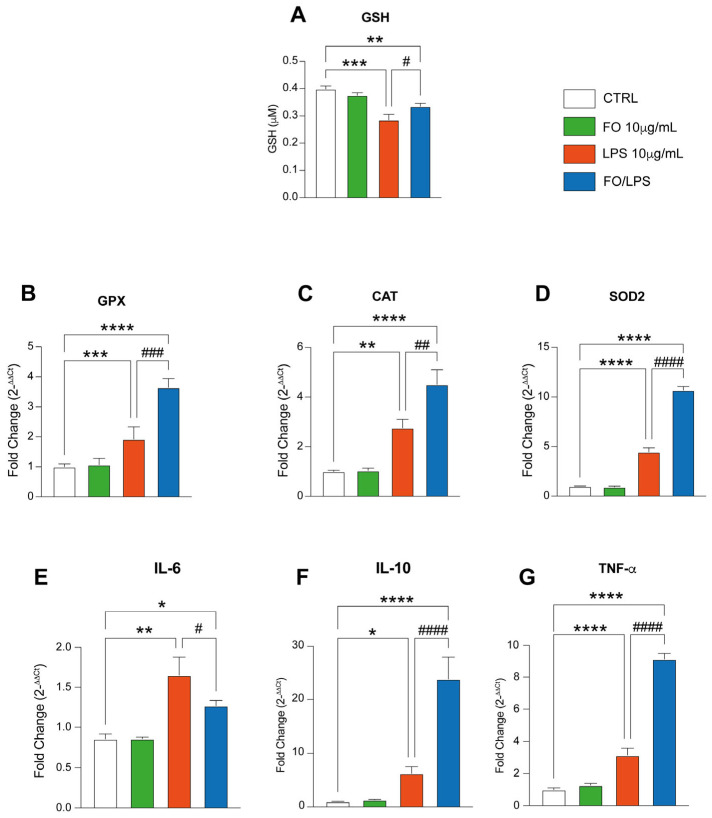
FO recovers cellular ROS scavenging ability. (**A**) FO supplementation implement GSH production upon LPS treatment. Histogram showing GSH quantification in BEAS following treatment with FO, LPS, and their combination. RT-PCR evaluating the expression GPX (**B**), CAT (**C**), SOD2 (**D**), IL-6 (**E**), IL-10 (**F**), and TNF- α (**G**) following treatment with FO, LPS, and their combination. Histograms are representative of 4 biological replicates. (* *p* ≤ 0.05; ** *p* ≤ 0.01; *** *p* ≤ 0.001; **** *p* ≤ 0.0001 compared to CTRL. # *p* ≤ 0.05; ## *p* ≤ 0.01; ### *p* ≤ 0.001; #### *p* ≤ 0.0001 compared to LPS).

To dissect the mechanism by which FO exerts its antioxidant effect, we sought to investigate the accumulation of different antioxidative markers. For this purpose, we started by assessing GSH protein accumulation following treatment with FO, LPS, and their combination. Interestingly, our analysis detected no significative change in GSH concentration following FO administration to our in vitro model (Figure 4A). However, LPS treatment dramatically decreases GSH accumulation compared to the untreated counterpart (Figure 4A). Interestingly, the FO/LPS combination completely recovers this condition, eventually showing a significative increase in comparison to the experimental condition where cells were treated with LPS. In comparison to untreated and FO-supplemented cells, however, GSH level are still decreased upon FO supplementation (Figure 4A). To further delve into the mechanism by which FO elicits its antioxidant effect, we decided to perform RT-PCR against some putative antioxidant genes. Interestingly, our data show no significative change in the expression of GPX, CAT, and SOD 2 expression following FO supplementation, thereby corroborating the previous data and proving that FO per se did not alter the BEAS antioxidative profile (Figure 4B–D). LPS treatment, on the other hand, triggered an enhancement of GPX, CAT, and SOD2 expression compared to the untreated counterpart. Notably, the pretreatment with FO led to a significative increase in the antioxidant defenses as justified by the enhancement of GPX, CAT, and SOD2 expression (Figure 4B–D). 

Furthermore, LPS interacts with TLR4 to induce cellular inflammatory response, thereby triggering cell oxidative stress enhancement and eventually apoptosis. Therefore, given the data we collected so far, we were prompted to investigate if FO might also reduce LPS-mediated inflammation. To dissect this point, we sought to assess the expression of some of the main inflammatory markers as IL-6, IL-10, and TNF- α (Figure 4E–G). Our data show that FO supplementation does not affect the expression of these markers in comparison to the untreated counterpart (Figure 4E–G). LPS supplementation, on the contrary, boosts the expression of IL-6, IL-10, and TNF- α (Figure 4E–G). Interestingly, the pretreatment with FO significatively decreases the expression of IL-6, but not of IL-10 and TNF- α, which were both enhanced following the treatment in comparison to the LPS-treated and control counterparts (Figure 4E–G). Overall, these data show evidence about the role excreted by FO in enhancing cellular antioxidant defenses and its involvement in cellular anti-inflammatory response.

### 2.4. FO Supplementation Recovers LPS-Mediated Inflammation by Boosting Pro-Resolving Mediators’ Pathway

**Figure 5:**
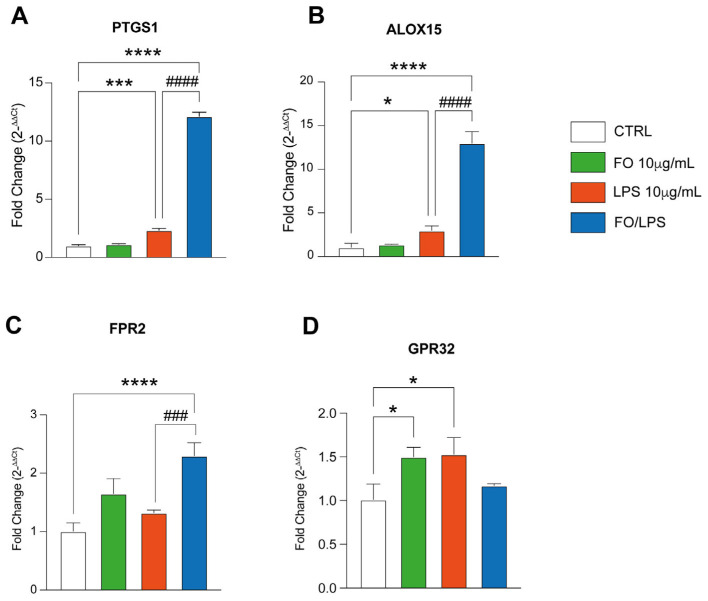
FO effect relies on resolvin-mediated inflammation resolution. RT- PCR analysis showing PTGS1 (**A**), ALOX15 (**B**), FPR2 (**C**), and GPR32 (**D**) expression following BEAS supplementation with FO, LPS, and their combination. Histograms are representative of 4 biological replicates. (* *p* ≤ 0.05; *** *p* ≤ 0.001; **** *p* ≤ 0.0001 compared to CTRL. ### *p* ≤ 0.001; #### *p* ≤ 0.0001 compared to LPS).

In order to dissect the mechanism underneath FO-mediated inflammation attenuation, we sought to investigate the expression of different genes involved in the pro-resolving mediated pathway. The latter has been reported to be derived from essential fatty acids to serve as a novel class of immunoresolvents, eventually limiting the acute response and orchestrating inflammation clearance [24]. Therefore, we first assessed the impact of FO per se on the expression of different genes involved in this pathway. Of note, our data show no significative change in the expression of PTGS1, ALOX15, and FPR2 in BEAS treated with FO compared to the control counterpart (Figure 5A–D). GPR32 expression, on the other hand, was significatively increased upon 24 h treatment with FO. Interestingly, inflammation induction by LPS supplementation, after 24 h, did not alter the expression of FPR2, eventually showing a marked increase in PTGS1, ALOX15, and GPR32 compared to the untreated counterpart (Figure 5A–D). Most importantly, after 24 h, the pretreatment with FO boosts the expression of all the markers, besides GPR32, eventually also showing a marked increase in comparison to the experimental condition in which BEAS cells were treated only with LPS (Figure 5A–D). Taken together, these data show that FO exerts its inflammatory clearance ability by boosting pro-resolving mediated pathway.

## 3. Discussion

In recent years, there has been a growing trend in the use of FO supplements, primarily due to their high content of omega-3 polyunsaturated fatty acids, namely EPA and DHA [25,26]. Interestingly, while mammals have the capability to biosynthesize these fatty acids from dietary precursors and essential fatty acids such as alpha-linolenic acid, this biosynthesis pathway entails a series of elongation and desaturation steps. Consequently, direct intake from supplements has emerged as a more efficient route of assimilation, leading to a significant surge in omega-3 supplement sales in recent years.

Several studies have delved into the effects of fish oil on oxidative stress and inflammation, revealing both favorable outcomes and potential concerns [27,28,29]. Omega-3 fatty acids present in fish oil are renowned for their antioxidant properties, which aid in reducing oxidative stress by neutralizing reactive oxygen species (ROS) [30]. These fatty acids can modulate signaling pathways involved in antioxidant defenses, such as the Nrf2 pathway, thereby enhancing the body’s ability to manage oxidative stress [31,32,33]. Given that these diseases are often characterized by heightened inflammation, the benefits of fish oil are closely linked to inflammation resolution [19].

Numerous studies have demonstrated the promise of fish oil in managing various lung diseases, owing to its anti-inflammatory and antioxidant properties [34,35,36,37]. Particularly, the beneficial effects of dietary fish oil rich in omega-3 have been evidenced in respiratory conditions such as COPD, asthma, and pulmonary distress, all characterized by a marked increase in inflammation [34,38].

Our study aimed to elucidate the cellular mechanism by investigating how fish oil functions in an in vitro model of lung inflammation using LPS on a healthy human bronchial epithelium cell line. 

Our data indicate that LPS administration for 24 h not only reduced cell viability but also elevated reactive oxygen species, depleted GSH, and induced mitochondrial depolarization, a marker of mitochondrial function impairment. The ensuing activation of the endogenous antioxidant system, including GPX, CAT, and SOD2, alongside inflammation markers such as IL-6, IL-10, and TNF-α, corroborates the induction of inflammation and oxidative stress by LPS in bronchial epithelial cells [37].

Our findings demonstrate that fish oil administration significantly mitigated ROS production, GSH depletion, and mitochondrial depolarization. Changes in the levels of GSH in the lung have been shown in various inflammatory conditions such as the epithelial lining fluid of the lower respiratory tract in idiopathic pulmonary fibrosis, ARDS, and cystic fibrosis [39,40,41]. Mitochondrial depolarization refers to impaired oxidative phosphorylation and energy production, a decline in mitochondrial membrane potential, and the increased production of ROS [42]. Mitochondria are crucial for cellular homeostasis and bioenergetics, whereas mitochondrial dysfunction has emerged as a key factor in the pathogenesis of human lung disease [43,44]. 

This effect is correlated with an upregulation in the expression of the endogenous cellular antioxidant system, as evidenced by the restoration of GSH levels and an increase in the gene expression of GPX, CAT, and SOD2, which appear to alleviate LPS-induced oxidative stress. Additionally, fish oil administration exhibited the suppression of IL-6 expression and a significant increase in the anti-inflammatory cytokine IL-10. Moreover, our data show a significant increase in TNF-α expression following FO pre-treatment. Increased IL-6 gene expression has been observed in epithelial cells from bronchial biopsy specimens of asthmatic subjects, and increased IL-6 levels are found in the bronchoalveolar lavage (BAL) fluid of symptomatic respiratory inflammation subjects [45]. IL-10 is an important agent in the resolution of inflammation. It acts as a cytokine synthesis inhibitory factor, displaying an ability to inhibit IFN-γ and IL-2 production in Th2 cells. IL-10 production and release by cells in response airway inflammatory diseases is upregulated by TNF-α and by the negative feedback regulation of itself [46]. Of note, TNF-α has been reported to also act as an inhibitor of IL-12 release by macrophages, indicating a mechanism for anti-inflammatory activity, which might be enhanced by FO administration [47].

The chemical characterization of the fish oil used in our study revealed the presence of pro-resolving mediators such as 17-Hydroxydocosahexaenoic acid (17-HDHA), 18-HEPE, and 14-HDHA. 17-HDHA, also known as Resolvin D1, and 14-HDHA are specialized pro-resolving lipid mediators derived from the omega-3 fatty acid DHA. It has been demonstrated that Resolvin D1 can attenuate neutrophil infiltration, reduce pro-inflammatory cytokines, and enhance alveolar macrophage function in ARDS and acute lung injury, while 14-HDHA has shown efficacy in reducing inflammation in the lungs by decreasing inflammatory cytokines such as TNF-α, IL-6, and MCP-1, which are crucial markers of lung inflammation [48,49].

18-HEPE is a specialized pro-resolving mediator (SPM) derived from EPA, and several studies have shown that 18-HEPE can modulate the inflammatory response in the lungs by enhancing the resolution phase, thereby reducing tissue damage and promoting recovery [50,51].

Our data indicate that the essential fatty acids DHA and EPA, along with the pro-resolving mediators contained in the fish oil used, were able to activate PTGS1 and ALOX15. The latter catalyzes the oxygenation of polyunsaturated fatty acids (PUFAs) to form hydroperoxides, which are subsequently converted into various bioactive lipids. The enzyme preferentially oxygenates arachidonic acid (AA) to 15-hydroperoxyeicosatetraenoic acid (15-HPETE), which is further metabolized to lipoxins. However, in the presence of EPA and DHA, it produces 18-HEPE, 17-HDHA, and 14-HDHA [52].

These enzymes, utilizing EPA and DHA as substrates, have been shown to produce pro-resolving mediators, which are metabolites of omega-3 which are responsible for inflammation resolution. Consistent with this, our results demonstrate an overexpression of the genes for FPR2 and GPR32, which are receptors of resolvins. 

It has been demonstrated that, in LPS-induced acute respiratory distress syndrome, pro-resolving mediators can stimulate alveolar fluid clearance and suppress polymorphonuclear leukocyte infiltration via FPR2 receptor signaling to mitigate pulmonary edema and lung injury [53,54]. Furthermore, Resolvin D1 can directly bind to FPR2 with higher affinity than GPR32, indicating a specific and potent anti-inflammatory pathway through FPR2 signaling [55,56]. 

Our data are in line with numerous studies that have demonstrated the effect of these molecules on inflammation resolution, and it has been observed that individuals often suffer from diseases characterized by chronic inflammation in the absence of omega-3 nutritional deficiency. 

## 4. Materials and Methods

### 4.1. Cell Culture and Treatments

BEAS cells were cultured in BEBMTM Basal Medium (Lonza Bioscience, Morrisville, NC, USA) supplemented with BPE, 2.00 mL, Insulin, 0.50 mL, Hydrocortisone, 0.50 mL, GA-1000, 0.50 mL, Retinoic Acid, 0.50 mL, Transferrin, 0.50 mL, Triiodothyronine, 0.50 mL, Epinephrine, 0.50 mL, and hEGF, 0.50 mL (Lonza Bioscience). At 80% confluency, the cells were treated with a trypsin-EDTA solution (0.05% trypsin and 0.02% EDTA), and the resulting cell suspension was adjusted to a cell density of 5 × 104 cells/mL. The cell suspensions were washed with a phosphate-buffered saline (PBS), resuspended in BEGMTM Bronchial Epithelial Cell Growth Medium, and placed in an incubator at 37 °C.

### 4.2. Fish Oil Characterization

Fish oil, supplied by Metagenics (Milan, Italy), is derived from sustainably caught sardines, herring, and small bluefish from the South Pacific region. The Certificate of Analysis was prepared by a lab holding ISO 17025 [57] Accreditation utilizing the analytical methods specified in GOED’s Technical Guidance Documents/Fatty Acid Analysis Methods [58]. The method recommended by GOED utilizes calibration specifications suitable for the analysis of EPA and DHA in concentrated oils. The results of analysis are shown in Table 1.

### 4.3. Cell Treatment and Viability Assay

To assess cell viability, we performed a 3-(4,5-dimethylthiazol-2-yl)-2,5-diphenyltetrazolium bromide (MTT) assay. Briefly, once a sufficient number of cells was obtained, cultures were maintained in FBS-free medium supplemented with 1% *v/v* bovine serum albumin (BSA, Sigma Aldrich, Milan, Italy), which was used as a vehicle in this experimental design. Subsequently, cultures were divided into groups to identify any potential toxicity of different concentrations of fish oil (FO) and lipopolysaccharide (LPS, Sigma Aldrich, Milan, Italy).

FO toxicity was assessed at 10, 50, and 100 μg/mL of FO after 24 h, while 1 and 10 μg/mL of LPS was assayed 1, 3, 24, and 48 h post-treatment. For this purpose, we employed the MTT assay. To perform this assay, cells were incubated with MTT salts for 3 h at 37 °C, dimethyl sulfoxide (DMSO, Sigma Aldrich, Milan, Italy) was then added to all wells, and readings were taken with Synergy H1 (Bio-Tek, Milan, Italy) at λ = 570 nm.

Experiments were performed by incubating the FO treatment for 24 h and then replacing it with fresh medium containing FO and LPS for the other 24 h. In the FO group, the fresh medium was replaced after 24 h with a new FO treatment.

### 4.4. GSH Quantification

The concentration of reduced glutathione (GSH) within cells was determined employing a colorimetric approach [59]. This entailed the interaction between free thiol groups and the 5,5-dithio-bis-(2-nitrobenzoic acid) (DTNB) reagent (Sigma Aldrich, Milan, Italy). Briefly, the cells were cultured in T25 flasks. Subsequently, the treatments were trypsinized, harvested, and centrifuged at 1500 rpm for 5 min at 4 °C. The cell pellets were then lysed by sonication and centrifuged again at 12,000 rpm for 15 min at 4 °C. Finally, 20 µL of the supernatant was added to the DTNB solution, and, after waiting for 20 min, we assessed the absorbance at λ = 412 nm (with a molar absorbance coefficient, εM, of 13,600 M^−1^∙cm^−1^). Each evaluation was carried out three times utilizing Synergy HT (Bio-Tek, Milan, Italy).

### 4.5. Mitochondrial Polarization Assessment

We evaluated alterations in mitochondrial membrane potential (ΔΨm) using the lipophilic dye JC-1 (Sigma Aldrich, Milan, Italy), [22]. This dye enters mitochondria and displays a reversible shift in fluorescence from green to red depending on mitochondrial membrane polarization. In healthy, viable cells with normal membrane potential, JC-1 forms dimers on the mitochondrial membrane, resulting in red fluorescence. Conversely, in damaged cells with altered membrane potential, JC-1 remains in the cytoplasm in a monomeric form, emitting green fluorescence. Cells were treated accordingly, incubated with JC-1 in darkness for 20 min at 37 °C, and then washed with phosphate-buffered saline (PBS) and analyzed with Synergy HT (Bio-Tek, Milan, Italy) at λ = 585 nm (red fluorescence = high value of ∆ψ) and 527 nm (green fluorescence = low value of ∆ψ).

### 4.6. ROS Measurement

The extracellular formation of reactive oxygen species (ROS) was determined fluorimetrically using a Synergy HT (Bio-Tek, Milan, Italy) (https://doi.org/10.3390/biom11060917). Briefly, 100 μL of cellular supernatant was added to each well of a 96-well microplate, and dichlorofluorescein diacetate (DCFH-DA D6883) (Sigma Aldrich, Milan, Italy), dissolved in dimethyl sulfoxide (DMSO, Sigma Aldrich, Milan, Italy), was added to a final concentration of 10 μM. The plate was then incubated for 1 h in the dark, and fluorescence was measured with excitation at λ = 485 nm and emission at λ = 528 nm.

### 4.7. Real-Time PCR

To measure gene expression, total RNA extraction was performed using Trizol (Life Technologies, Milan, Italy). The extracted mRNA was then converted into cDNA through reverse transcription using the High-Capacity cDNA Reverse Transcription Kits (Thermo Fisher Scientific, Waltham, MA, USA). Quantitative analysis was carried out using SYBR Green PCR MasterMix (Life Technologies, Milan, Italy) and primers for the following genes: Glutathione Peroxidase (GPX), Catalase (CAT), Superoxide Dismutase 2 (SOD2), Interleukin-6 (IL-6), Interleukin-10 (IL-10), Tumor necrosis Factor alpha (TNF-α), Prostaglandin-Endoperoxide Synthase 1(PTGS1), Arachidonate 15-Lipoxygenase (ALOX15), G Protein-Coupled Receptor 32 (GPR32), and Formyl Peptide Receptor 2 (FPR2) using the Rotor-Gene Q (Qiagen, Hilden, Germany) instrument. For each sample, gene expression levels were normalized using β-actin expression levels. The primers are listed in Table 2. The relative mRNA expression level was calculated using the comparative 2^−ΔΔCt^ method.

### 4.8. Statistical Analysis

To compare three or more groups, we employed one-way analysis of variance (ANOVA) followed by the Bonferroni post hoc test for multiple comparisons. Data are expressed as the mean ± SD of 4 biological replicates. GraphPad Prism software, version 5.00, was utilized for data analysis. Statistical significance was defined as *p* < 0.05 (symbols indicating statistical differences are detailed in the figure legends).

## 5. Conclusions

The findings of this study are consistent with the growing body of literature highlighting the therapeutic potential of omega-3 fatty acids in managing inflammatory conditions, particularly those affecting the respiratory system. The administration of fish oil in our in vitro model of lung inflammation induced by LPS showed significant mitigation of oxidative stress markers, including ROS production, GSH depletion, and mitochondrial depolarization. This suggests that fish oil not only acts as an anti-inflammatory agent, but also enhances cellular antioxidant defenses. Collectively, the results of this study underscore the importance of a diet rich in omega-3 fatty acids and suggest that fish oil could be utilized as a dietary supplement to prevent pulmonary diseases characterized by unresolved inflammation.

## Figures and Tables

**Table 1:** Fish oil characterization.

Determinations	Specification	Result
**Fatty Acid Profile**		
EPA mg/g (as FFA)	50–150	101.0
DHA mg/g (as FFA)	100–225	175.8
Total Omega-3 mg/g (as FFA) *	250–425	325.1
* (Sum of 18:3 w3, 18:4 w3, 20:4 w3, 20:5 w3, 21:5 w3, 22:5 w3, 22:6 w3)		
**SPM Content**		
17-HDHA as FFA (mg/kg)	40–200	137.7
18-HEPE as FFA (mg/kg)	25–200	97.5
14-HDHA as FFA (mg/kg)	20–100	22.7
**Analytical Data**		
Acid Value (mg KOH/g)	Max. 3	0.47
Peroxide Index (meq O_2_/kg)	Max. 5	2.38 (at time of release)
Anisidine value	Max. 20	11.0 (at time of release)
Weight Variation (Upon Fill)	Avg. 10%	Complies
Disintegration time (min)	Max. 30	7
**Contaminant Data**		
Arsenic (mg/kg)	Max. 1	<0.05
Cadmium(mg/kg)	Max. 1	<0.01
Lead (mg/kg)	Max. 0.33	<0.05
Mercury (mg/kg)	Max. 0.1	<0.01
**Microbiological parameters**		
Aerobic Plate Count (CFU/g)	Max. 1000	<10
Yeast and Mould (CFU/g)	Max. 100	<10
*S.* *aureus*	Negative/10 g	Negative
Salmonella	Negative/10 g	Negative
*E. coli*	Negative/10 g	Negative

**Table 2:** Primers’ list.

Primer	Forward	Reverse
GPX	ACAAGAACGGCTGCGTGGTGAA	GCCACACACTTGTGGAGCTAGA
CAT	GTGCGGAGATTCAACACTGCCA	CGGCAATGTTCTCACACAGACG
SOD2	CTGGACAAACCTCAGCCCTAAC	AACCTGAGCCTTGGACACCAAC
IL-6	AGACAGCCACTCACCTCTTCAG	TTCTGCCAGTGCCTCTTTGCTG
IL-10	TCTCCGACAAGGCTTGGCAACCCA	TCAGACAAGGCTTGGCAACCCA
TNF-a	CTCTTCTGCCTGCTGCACTTTG	ATGGGCTACAGGCTTGTCACTC
PTGS1	GATGAGCAGCTTTTCCAGACGAC	CCAGACTGGATGCAGGACACAA
ALOX15	ACCTTCCTGCTCGCCTAGTGTT	GGCTACAGAATGACGTTGGC
FPR2	GCCTTTTGGCTGGTTCCTGTGT	CCAGACTGGATGCAGGACACAA
GPR32	GTGATCGCTCTTGTTCCAGGAAG	TGCGTGCCATACGGAAGACAGT
b-Actin	CACCATTGGCAATGAGCGGTTC	AGGTCTTTGCGGATGTCCACGT

## Data Availability

The original contributions presented in the study are included in the article, further inquiries can be directed to the corresponding author.
